# Raman Fingerprint of Extracellular Vesicles and Conditioned Media for the Reproducibility Assessment of Cell-Free Therapeutics

**DOI:** 10.3389/fbioe.2021.640617

**Published:** 2021-04-13

**Authors:** Cristiano Carlomagno, Chiara Giannasi, Stefania Niada, Marzia Bedoni, Alice Gualerzi, Anna Teresa Brini

**Affiliations:** ^1^IRCCS Fondazione Don Carlo Gnocchi ONLUS, Milan, Italy; ^2^Department of Biomedical Surgical and Dental Sciences, University of Milan, Milan, Italy; ^3^IRCCS Istituto Ortopedico Galeazzi, Milan, Italy

**Keywords:** mesenchymal stem/stromal cells, Raman spectroscopy, extracellular vesicles, conditioned medium, secretome, orthobiologics

## Abstract

Extracellular Vesicles (EVs) and Conditioned Medium (CM) are promising cell-free approaches to repair damaged and diseased tissues for regenerative rehabilitation purposes. They both entail several advantages, mostly in terms of safety and handling, compared to the cell-based treatment. Despite the growing interest in both EVs and CM preparations, in the light of a clinical translation, a number of aspects still need to be addressed mainly because of limits in the reproducibility and reliability of the proposed protocols. Raman spectroscopy (RS) is a non-destructive vibrational investigation method that provides detailed information about the biochemical composition of a sample, with reported ability in bulk characterization of clusters of EVs from different cell types. In the present brief report, we acquired and compared the Raman spectra of the two most promising cell-free therapeutics, i.e., EVs and CM, derived from two cytotypes with a history in the field of regenerative medicine, adipose-derived mesenchymal stem/stromal cells (ASCs) and dermal fibroblasts (DFs). Our results show how RS can verify the reproducibility not only of EV isolation, but also of the whole CM, thus accounting for both the soluble and the vesicular components of cell secretion. RS can provide hints for the identification of the soluble factors that synergistically cooperate with EVs in the regenerative effect of CM. Still, we believe that the application of RS in the pipeline of cell-free products preparation for therapeutic purposes could help in accelerating translation to clinics and regulatory approval.

## Introduction

Over the years, mesenchymal stem/stromal cells (MSCs) have gained popularity as therapeutics in a variety of clinical scenarios thanks to their ability to promote tissue regeneration and reduce inflammation. Among the most common MSC harvesting sources, the stromal vascular fraction of adipose tissue stands out as one of the most convenient in terms of both harvesting procedure and cell yield (Chu et al., 2019). To date, the clinical studies relying on the use of Adipose-derived MSCs (ASCs) comprise different applications, ranging from musculoskeletal diseases such as osteoarthritis to diabetes mellitus, colitis, and autoimmune disorders (Chu et al., 2019). Of note, in recent months ASC administration has also been evaluated as a therapeutic strategy for the treatment of Acute Respiratory Distress Syndrome in COVID-19 patients (Rogers et al., 2020).

Also recently, the therapeutic potential of dermal fibroblasts (DFs) has attracted scientific interest, mainly in the field of wound healing and skin grafts (Ichim et al., 2018). While the most common therapeutic applications include the treatment of acute and chronic wounds, burns, epidermolysis bullosa, and ulcers, we recently gave evidence also of a pro-osteogenic potential of DF secretome (Niada et al., 2018).

ASCs and DFs share common characteristics (such as immunophenotypic profile and differentiative potential) and, when implanted at the damaged site, both of them are able to exert immunomodulatory and regenerative actions (Nilforoushzadeh et al., 2017; Ichim et al., 2018).

Despite the overall success obtained in clinical trials, cell therapy presents several challenges, such as safety/regulatory concerns and technical aspects (harvesting procedure, cell expansion, and storage of the final product). In the past decade, increasing evidence has led to a paradigm shift in the mechanism of action of cell-based therapies, from the initial belief in a direct replacement of the damaged tissue to the evidence of paracrine signaling orchestrating the regenerative process. In comparison with cell-based strategies, cell-free approaches entail several advantages, mostly in terms of safety and handling. Nevertheless, in the light of a clinical translation a number of aspects still need to be addressed.

Extracellular vesicle-related (EV) research and application have attracted considerable commercial interest and investment thanks to their potential in diagnosis and therapy. In the field of regenerative medicine and regenerative rehabilitation (Willett et al., 2020), EVs released by MSCs have demonstrated their ability to foster recovery and repair of damaged and aged tissues. Despite the proven advantages, application methods and efficacy are debated mainly because of limits in the reproducibility and reliability of the proposed protocols. Indeed, the technical challenge is still open as no appropriate and quantifiable performance metrics have been developed yet to objectively assess the repeatability and efficiency of the multitude of suggested methods for separation and isolation of EVs from an MSC-derived culture medium. Therefore, data interpretation and assessment of EV treatment efficacy in regeneration studies are difficult to achieve.

Since their discovery as therapeutic agents, the EV efficacy as cell-free therapy has been compared to that of a conditioned medium (CM), which represents a mixture of different factors secreted by the cells, including growth factors and cytokines, enzymes, nucleic acids, bioactive lipids, and of course, EVs. From this perspective, the potential of MSC-derived EVs and CM as innovative biological approaches for the treatment of osteoarthritis is currently a hot topic in the orthopedic field (D’Arrigo et al., 2019). Although the production of CM preparations is simple in principle, their characterization and standardization are a major issue that needs to be overcome to deliver clinically acceptable products. Up to now, CM preparations are mainly analyzed for the presence of pro-inflammatory factors (Sriramulu et al., 2018), while EV preparations are commonly characterized by the size and number of particles, presence of EV-specific surface markers, and total protein concentration (Thery et al., 2018). Conversely, the correlation between the folds of concentration (routine CM concentrates) or protein concentration/number of particles (EV preparations) and product safety and potency is extremely limited or not even addressed (Bogatcheva and Coleman, 2019).

EVs are a subproduct of CM preparations. For this reason, it is important to consider that isolated EV populations can vary significantly depending on the isolation method considered and/or concentration procedure. Distinct isolation methods can lead to EV products with different purity levels, EV dimensions, intracellular origin, and thus with heterogeneous biochemical composition, and possibly regenerative effects. Differences in isolation procedures have brought confusing results and misleading opinions about EV application in regenerative medicine.

Raman spectroscopy (RS) is a non-destructive vibrational investigation method that can provide detailed information about the biochemical composition of a sample by taking advantage of the vibration modes of the chemical bonds present within molecules irradiated by laser. The collected spectrum is a combination of signals provided by lipids, proteins, nucleic acids, and metabolites in relation with their presence, concentration, coordination, modifications, interactions, and environment. The Raman bulk characterization of clusters of MSC-derived EVs can help assessing their purity and effective isolation (Gualerzi et al., 2019). Moreover, RS distinguishes with high accuracy EVs derived from different cell types (Gualerzi et al., 2017). For the present brief report, we acquired and compared the Raman spectra of two promising cell-free therapeutics, i.e., EVs and CM, derived from two cytotypes with a history in the field of regenerative medicine, ASCs and DFs. Our results show how RS can verify the reproducibility not only of EV isolation from ASCs and DFs, but also of an even more complex type of sample, the whole CM, accounting for both the soluble and the vesicular components of cell secretion. Moreover, RS can provide hints for the identification of the soluble factors that synergistically cooperate with EVs in the regenerative effect of CM.

## Materials and Methods

### Cell Isolation and Maintenance

ASCs and DFs were isolated from the waste tissues collected at IRCCS Istituto Ortopedico Galeazzi under the Institutional Review Board approval. ASCs were derived from two male and four female donors (mean age 44 ± 16 y/o) undergoing aesthetic (*n* = 4) or prosthetic (*n* = 2) surgery, following well-established protocols (de Girolamo et al., 2009). Briefly, the subcutaneous adipose tissue was fragmented with a scalpel and digested with 0.75 mg/ml type I Collagenase (Worthington Biochemical Corporation, Lakewood, NJ, United States) for 30 min at 37°C. DFs were derived from three female donors (mean age 39 ± 12 y/o) undergoing aesthetic surgery, following standard protocols (Niada et al., 2018). Briefly, the abdominal dermis was first subjected to a mechanical fragmentation and then enzymatically digested with 0.1% type I collagenase for 30 min at 37°C. Isolated ASCs and DFs were then plated at the density of 10^5^ cells/cm^2^ in high glucose DMEM plus 10% FBS (EuroClone, Pero, Italy), 2 mM l-glutammine (Sigma-Aldrich, St. Louis, MO, United States), 50 U/ml penicillin and 50 μg/ml streptomycin (Sigma-Aldrich, St. Louis, MO, United States) and expanded for IV-VI passages for EV and CM production.

### EV Isolation and CM Production

Conditioned media were collected from 80 to 90% confluent ASCs and DFs cultured for 3 days in starving conditions (absence of FBS). Supernatants were first centrifuged at 2,500 g for 15 min at 4°C to remove dead cells, large apoptotic bodies, and debris, and then they followed different routes in order to isolate EVs rather than obtain CM. EV isolation was performed through differential centrifugation at 100,000 g in a SW 41 Ti swinging-bucket rotor (Beckman Coulter, Brea, CA, United States) (Gualerzi et al., 2017) while CM concentration was achieved by spinning the samples for 90 min at 4,000 g through Amicon Ultra-15 Centrifugal Filter Devices with 3 kDa cut-off (Merck Millipore, Burlington, MA, United States) (Niada et al., 2018).

### Nanoparticle Tracking Analysis

For each cell type, coupled EV and CM samples were diluted in 0.22 μm triple-filtered PBS and the nanoparticle tracking analysis (NTA) was performed by NanoSight NS300 (Malvern PANalytical, Salisbury, United Kingdom). Each measurement consisted in three videos lasting 1 min. All captures complied with the quality criteria of 20–120 particles/frame, concentration ranging from 10^6^ to 4 × 10^9^ particles/ml and valid tracks > 20%. Data analysis was performed with the in-build NanoSight Software NTA.

### Western Blot

Prior to Western Blot analysis, an aliquot of each CM sample was quantified through the Bio-Rad Protein Assay (Bio-Rad, Milan, Italy), while EV pellets were directly lysed in the appropriate buffer without protein quantification (Niada et al., 2020). The amount of 10 μg of CM, corresponding to ∼25 μl, and EVs derived from 1.5 × 10^6^ ASCs or DFs were lysed in 5% 2-Mercaptoethanol and 2X Laemmli Buffer (Bio-Rad, Milan, Italy), separated in a 4–15% polyacrylamide gel (Bio-Rad, Milan, Italy) and transferred to a nitrocellulose membrane (GE Healthcare, Chicago, IL, United States) (Niada et al., 2020). After being blocked with 5% non-fat dried milk (AppliChem, Darmstadt, Germany) and 0.1% Tween (Promega, Madison, WI, United States) in PBS, samples were probed overnight at 4°C for the expression of Alix (NBP1-90201, 1:1,000 diluted, Novus Biologicals, Centennial, CO, United States), FLOT-1 (1:500 diluted, BD Transduction Laboratories, San Jose, CA, United States), TSG101 (T5701, 1:1,000 diluted, Millipore, Burlington, MA, United States), and CD9 (1:1,000 diluted, System Biosciences, Palo Alto, CA, United States). All washing steps were performed with 0.1% Tween in PBS, and after incubation with appropriate peroxidase-conjugated secondary antibodies (sc-2004, 1:5,000 diluted, Santa Cruz Biotechnology, Dallas, TX, United States and 62-6520, 1:20,000 diluted, Thermo Fisher Scientific, Waltham, MA, United States and System Biosciences, Palo Alto, CA, United States), bands were revealed using ECL (Cyanagen, Bologna, Italy). Images were acquired with the ChemiDoc imaging system (Bio-Rad, Milan, Italy).

### Raman Spectroscopy

Samples were analyzed by RS following a previously reported protocol for the bulk characterization of EVs (Gualerzi et al., 2017, 2019). Briefly, 5–10 μl of EV suspension or CM were deposited on a calcium fluoride slide and air-dried. Measurements were performed with Raman microspectroscopy (LabRAM Aramis, Horiba Jobin Yvon S.A.S., Lille, France) equipped with a 532 nm laser and with a 50× objective (N.A. 0.75), 1,800 grooves/mm diffraction grating, 400 μm entrance slit, and confocal mode (300 μm pinhole) in the spectral ranges 600–1,800 and 2,600–3,200 cm^–1^. Calibration was performed using silicon reference peak (520.7 cm^–1^). Taking advantage of the integrated software Labspec 6 (Horiba Jobin Yvon S.A.S., Lille, France), we performed a baseline correction (fourth-degree polynomial curve), unit vector normalization, and post-acquisition calibration before the statistical analysis of spectra.

### Statistical Analysis

Descriptive and multivariate statistical analysis of Raman spectra were performed by Origin 2018 (OriginLab, Northampton, MA, United States) as previously described (Gualerzi et al., 2017, 2019). Principal component analysis (PCA) of the normalized and aligned spectra was performed and followed by linear discriminant analysis (LDA). The classification model created, based on leave-one-out cross-validation, allowed us to evaluate the discrimination power between cell sources of EVs and CM. The non-parametric Kruskal-Wallis test was then performed on canonical variable scores to verify that the means of each group were significantly different, despite within-group variance. The Kruskal-Wallis test was performed also to evaluate the inter-donor variability of canonical variable 1 and 2 scores, with a *p*-value of 0.05.

## Results

EV and CM preparations were obtained in conformity with previously reported protocols with tested efficacy in regeneration studies. To compare the two cell-free preparations, size distribution and concentration of particles were assessed.

**FIGURE 1:**
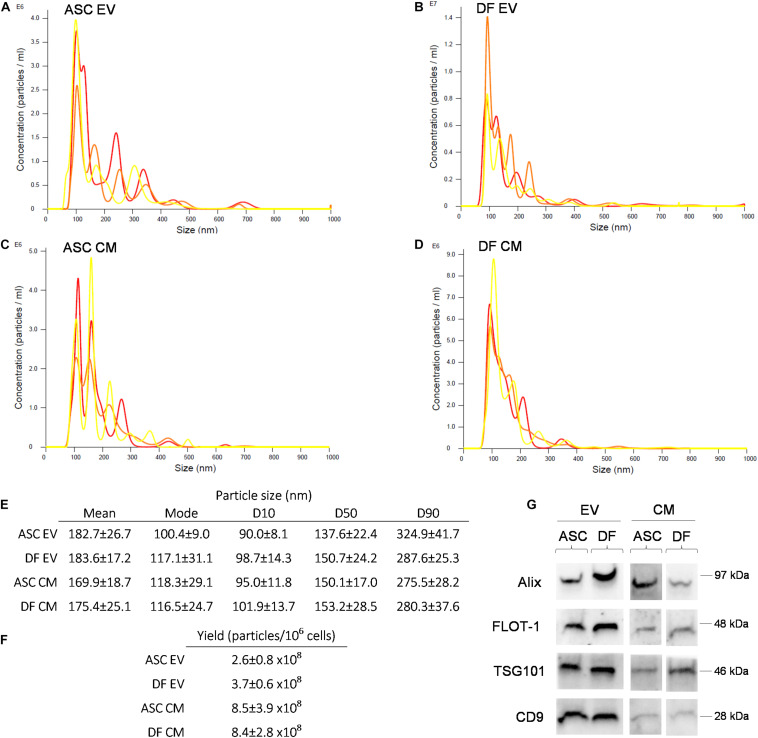
**(A–D)** Representative NTA of EV and CM samples derived from ASCs and DFs. Each graph shows the size distribution of 3 consecutive 1 min runs for each sample. **(E–F)** Size distribution and vesicular yield deriving from 6 NTA measurements/group shown as mean ± SD. **(G)** Western Blot of CM and EV samples from ASCs and DFs, showing the expression of the vesicular markers Alix, FLOT-1, TSG101 and CD9. In each lane, 10μg of CM or EV deriving from 1.5 × 10^6^ cells were loaded.

NTA revealed a similar size distribution between all samples (Figures 1A–E), with 50% of the events falling inside the dimensional range of 150 nm. No significant difference was observed in size distribution between EV and CM samples (non-parametric Mann-Whitney test; *p* > 0.05). Given the same number of donor cells, the vesicular yield is comparable between the ASCs and DFs. As expected, EV preparations showed a lower number of particles/10^6^ cells in comparison with coupled CM samples. Indeed, the post-ultracentrifugation recovery was about 30% of the input for ASCs and 44% for DFs, as shown in Figure 1F. Besides, we obtained the purity score by calculating the ratio between the number of particles and the total protein content on the same sample (Gualerzi et al., 2019). The data demonstrated that EV preparations from both ASCs and DFs have higher purity scores compared to CM samples, as expected (data not shown). The expression of the canonical markers Alix, FLOT-1, TSG101, and CD9 was confirmed in all samples by Western Blot, as shown in Figure 1G.

**FIGURE 2:**
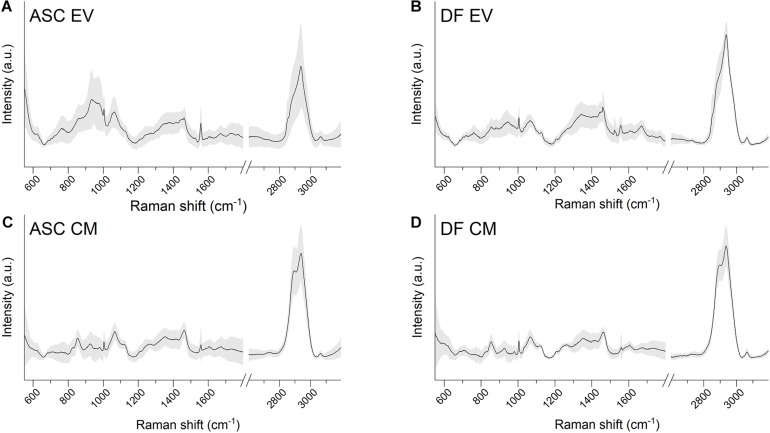
**(A–D)** Average Raman spectra obtained with 532 nm laser line on air-dried drops of EV or CM samples lied on calcium fluoride slides. Both ASC-derived and DF-derived samples are shown. Gray shaded areas represent ± 1 standard deviation.

Raman spectroscopy analysis of EV suspensions and CM preparations were performed on dried drops layed on Raman-transparent calcium fluoride slides. Data were obtained in the spectral range 600–1,800 and 2,600–3,200 cm^–1^ with a good signal-to-noise ratio and a good reproducibility of the spectra in the acquisition conditions considered, as attested by the values of standard deviation (Figures 2A–D). Raman data were acquired for all of the EV and CM samples from both ASCs and DFs, obtained from the cells of six and three different donors, respectively.

The average spectra from both EVs and CM extracted from the identified cell sources showed the characteristic Raman bands of proteins (Amide I 1,650 cm^–1^), lipids (2,700–3,200 cm^–1^), and nucleic acids (720–820 cm^–1^). The EV average spectra are in agreement with previously reported bulk characterization of vesicles by RS (Gualerzi et al., 2017, 2019). Interestingly, the lipid content described by the CH, CH_2_, and CH_3_ bonds (in the spectral range 2,600–3,200 cm^–1^) was different between EV and CM samples, suggesting that the main differences between the two types of cell-free preparations might involve the lipid components.

**FIGURE 3:**
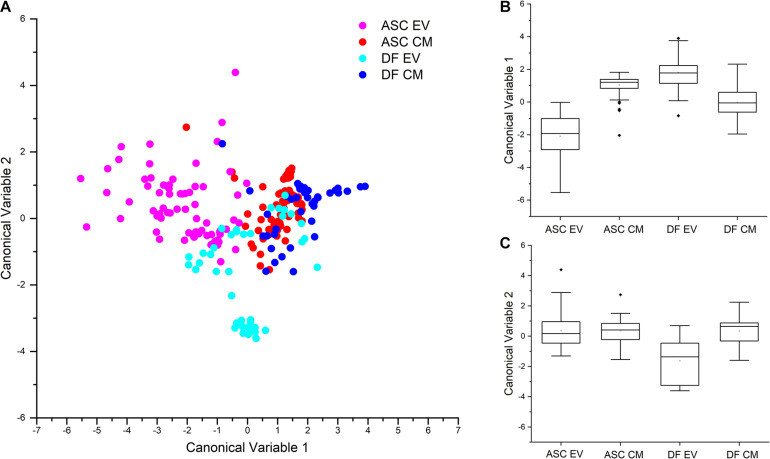
**(A)** Scatterplot reporting the results of the PCA-LDA multivariate statistical analysis of ASC- and DF-derived secretome products, EVs and CM. Each dot represents one spectrum that has been assigned with canonical variable 1 and canonical variable 2 scores. As reported in the legend, different colors are assigned to spectra based on the source: ASC-derived EVs, pink; ASC-derived CM, red; DF-derived EVs, light blue; DF-derived CM, blue. Partial overlap of blue and red dots shows similarities in the spectra from ASC- and DF-derived CM. B, C: Box plots representing the canonical variable 1 **(B)** and canonical variable 2 **(C)** scores obtained after PCA-LDA analysis and the respective results of the non-parametric Kruskal-Wallis test for the analysis of variance. The statistical data demonstrate that CM and EV preparations can be distinguished by the PCA-LDA classification model.

To further investigate the reproducibility of the acquisition setting and the differences in the secretome of the two cell sources, Raman data from both EVs and CM preparations were considered for multivariate PCA-LDA analysis. The scatterplot shown in Figure 3A graphically represents the results of the multivariate analysis of EV- and CM-derived spectra from ASC and DF samples, with each dot representing one single spectrum. In accordance with our previous data (Gualerzi et al., 2017), the significant differences observed in the spectral profiles of EVs from ASCs and DFs, determine an error rate in the classification of EV samples of 21.92% for ASC-derived EVs and of 2.38% for DF-derived EVs. The spectral difference between the two EV samples is visually represented in the scatter plot of Figure 3A: the dots corresponding to the Raman spectra of ASC-derived EVs (pink) have a minimal overlap with those from DF-derived EVs (light blue). Interestingly, also CM preparations from ASCs and DFs could be distinguished by means of the PCA-LDA classification model. Although spectral similarities between CM samples determine a partial overlap in the scatter plot reported in Figure 3A, the proposed classification model suggests the possibility to distinguish between the secretome of ASCs and that of DFs by their Raman profile. This result is in agreement with previously reported data from differential proteomic analysis performed on CM samples from ASCs and DFs, demonstrating that CM from these cell types share common proteomic patterns (Niada et al., 2018). Collectively, the mean values of canonical variable 1 obtained for ASC- and DF-derived samples were demonstrated to be significantly different (*p* < 0.001, Kruskal-Wallis test, Figure 3B) for both EVs and CM. To verify that the difference between CM and EV preparations was related to the cell source and not due to donor-associated differences, Kruskal-Wallis test was performed demonstrating that the canonical variable 1 and 2 scores were not significantly different between donors at the 0.05 level. On the contrary, looking at the canonical variable 2 values, only those from DF-derived EV samples were shown to be significantly different from DF-derived CM and ASC-derived EVs and CM (Figure 3C).

Looking at the classification error rate after leave-one-out cross-validation, the calculated mean percentage of misclassification was 25.43%, with the lower value of error rate for DF-derived EVs (14.29%).

**FIGURE 4:**
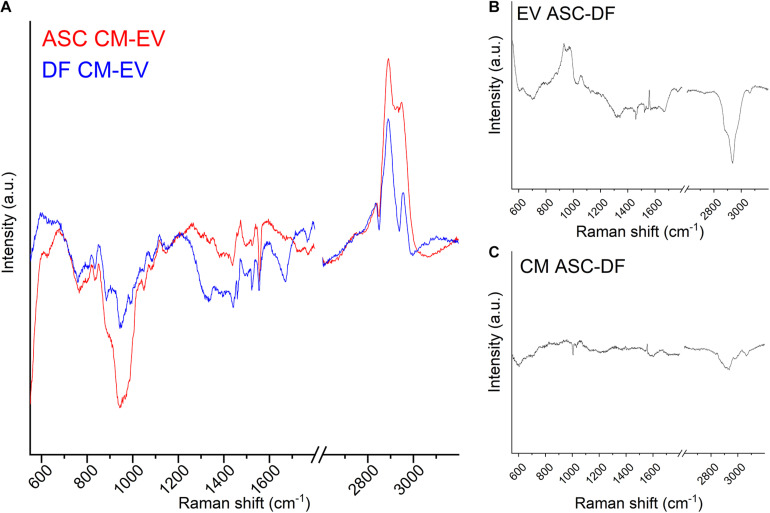
**(A)** Subtraction spectra obtained for ASC (red line) and DF (blue line) preparations by subtracting the EV average spectrum to the CM average spectrum. **(B,C)** Subtraction spectra obtained for CM **(B)** and EV **(C)** preparations by subtracting the ASC average spectrum to the DF average spectrum. All graphs display the same scale for the *y*-axis (Intensity of the Raman signal).

In order to deepen the reasons for the observed spectral differences, Figure 4A shows the subtraction spectra obtained by subtracting the EV average spectrum to the CM average spectrum for ASC (red line) and DF (blue line) preparations. We can speculate that the reported spectra represent mainly the contribution of soluble secretome factors to the CM samples, once the contribution of EVs was removed. Although PCA-LDA analysis highlighted partial overlap in the spectra from CM preparations, the subtraction of the EV spectral contribution made the differences between the non-EV secretome of ASCs and DFs apparent. It is worth noting that, in the present study, the non-EV secretome might include those particles that cannot be separated by the ultracentrifugation method due to poor yield and technical limitation (Thery et al., 2018). For this reason, we cannot exclude the contribution of a subpopulation of EVs, possibly both small and large EVs, to the reported spectral differences.

The main peaks of the subtraction spectrum were identified, revealing that the main differences can be attributed to the saccharide content of the samples (Movasaghi et al., 2007). In particular, 761–764 (Tryptophan ring or Pyrimidine ring); 832–836 (O-P-O stretching, Tyrosine/DNA); 941.6 (skeletal modes, polysaccharides); 1,049 (Glycogen); 1,440 (CH/CH_2_ deformation); 2,836–2,839 and 2,890.3 cm^–1^ (contributions from CH2 asymmetric stretch of proteins and lipids) were identified as prominent peaks in the subtraction spectra of both cell types, thus describing the major differences in the biochemical composition of CM compared to EVs for both cell sources. On the contrary, the 971 cm^–1^, attributed to the C-C stretching of proteins, was peculiar of ASC-derived data, whereas 2,930 and 2,949 cm^–1^ characterized the DF subtraction spectrum, underlying that lipids play a major role in the biochemical difference of cell-free preparations from this cell type.

Finally, we obtained the subtraction spectra for CM and EV preparations by subtracting the spectrum of the DF-derived samples to the ASC-derived one. Figures 4B,C show the results using the same scale on the *y*-axis, i.e., the same intensity scale. Spectral differences are more prominent between EV spectra than CM spectra from ASCs and DFs, suggesting that CM chemical composition and content are more similar than the EV cargo between the two cell sources. The subtraction spectra describe the differences highlighted by PCA-LDA from another point of view.

## Discussion

In the present work, we demonstrated that CM preparations, compared to EV ones, retained a 3–4 times higher number of particles per million donor cells. This result can be attributed to a suboptimal yield of the ultracentrifugation procedure, as already described in the literature (Tang et al., 2017; Takov et al., 2019). Nevertheless, the isolation procedure through ultracentrifugation did not affect EVs quality as far as size distribution and antigen profile are concerned.

The RS characterization of CM and EV preparations has demonstrated its usefulness in assessing the quality and repeatability of the cell-free product, but it has also brought to light biochemical similarities and differences between the two preparations. The present data confirm previously reported observations about the ability of RS to uncover the biochemical differences between EVs released by different cell sources (Gualerzi et al., 2017), as well as the possibility to use the spectroscopic method to highlight differences in the purity of EV samples obtained by different protocols (Gualerzi et al., 2019). Concerning this latter point, it was previously suggested that variations in the method of EV isolation influence the quality and quantity of co-isolated soluble factors (quantified also by the purity scores) and induce the selection of subpopulations of EVs. The data herein reported confirm the possibility of using Raman analysis to characterize EV products with variable purity index, but also different cell-free preparations, like CM samples. The advantage of the proposed methodology is related to the identification of a spectroscopic fingerprint that does not imply the detection and labeling of a single or a limited panel of specific antigens but provides an overall description of the content of the preparation that is under investigation. In the search for the optimal protocol for EV or CM preparation, the Raman analysis can verify the repeatability of the downstream product. Once the adequate translational procedure is found, the Raman data can verify the content of multiple batches, repeatedly, without the need for *ad hoc* preparations to be “sacrificed” for the quality check. Despite the fact that most researchers dealing with cell-free products for regenerative medicine might find the methodology apparently complicated and possibly expensive, it is worth noting that, once optimized, the acquisition protocol does not require sample preparation and it could be performed using portable, cost-effective instruments, commercially available and widely used for diagnostic purposes. Differently from other interesting approaches for EV detection that take advantage of Surface Enhanced Raman Scattering (SERS) (Lee et al., 2017; Chalapathi et al., 2020), the proposed protocol relies on the bulk characterization of the specimen, with less intense signals but more versatile. The SERS approach is able to highly enhance the Raman effect provided by various biological molecules present in a specific fluid, but the enhancement is not selective because it relies on the non-specific formation of a hot spot between the nanostructured metallic material and the molecule. In our case, the CM characterized present various heterogeneous biological molecules, and consecutively, the application of the SERS regimen could provide the enhancement of the Raman signal of unwanted molecules, e.g., proteins, masking the effective CM Raman signature.

As for the similarities in the spectral signature of CM and EV samples within the same cell source, we can speculate that some of them might be related to the particles present within the CM preparation. Nonetheless, it should be noted that other soluble factors could be shared between CM and EV samples, i.e., molecules co-isolated with vesicles or bound to the external surface of EVs. Another interesting aspect is the partial overlap of ASC- and DF-derived CM samples obtained by PCA-LDA analysis, revealing that the impact of the cell source is more evident for EV preparations. We hypothesize that this result may in part depend on the different route of secretion between soluble factors and EV cargo, the latter being more selectively controlled by the cells, especially for miRNA sorting (Abels and Breakefield, 2016). Moreover, EV lipid composition shares common features with the cell of origin. Therefore, even though CM samples represent a complex cocktail accounting for a lot more than EVs, the presence of common freely secreted molecules between ASCs and DFs may flatten the statistical comparison and mask the impact of the observed differences on EV composition. Of note, through a differential proteomic approach, we recently gave evidence of a slightly lower similarity between the CM samples from the two cell populations in comparison to EV ones (93.4% vs. 97.2% of shared proteins between CM and EVs, respectively) (Niada et al., 2020). This discrepancy is most probably due to the contribution of non-protein molecules to the Raman profile of the samples. Here we suggest that the factors that majorly contribute in distinguishing ASC- and DF-EVs are lipids rather than proteins, confirming previously reported data (Gualerzi et al., 2017). It also has to be noted that the lipid components involved in the biochemical differences between the considered samples comprise but are not limited to the EV lipid bilayer. Both EVs and CM might include bioactive lipids, like endocannabinoids, that can be freely secreted by the cell and mediate communication among different cell types. As a consequence, deep lipidomic analysis should be performed to investigate the origin of the observed spectral differences between the two preparations.

Moreover, the subtraction spectra between CM and EV revealed that, for both cell types, remarkable differences seem to be related to saccharide content. Considering that theoretically CM samples contain naïve vesicles, since the process to obtain them consists simply of a filtration step, we can hypothesize that ultracentrifugation may enrich an EV subpopulation with specific carbohydrate contents, e.g., peculiar glycosylation profiles. Indeed, in recent years EV glycomics has attracted scientific interest for its implications both as a diagnostic tool (Williams et al., 2018) and in the study of EV uptake (Williams et al., 2019). From this perspective, a first report on the impact of the isolation method on the EV glycosylation profile has been recently published (Freitas et al., 2019).

## Conclusion

In conclusion, with the present work we took a step forward in the characterization and molecular profiling of two secretome formulas derived from distinct cell sources by providing evidence of both a quantitative difference in the yield of vesicular elements per million cells between CM and EV preparations and a qualitative difference in the Raman spectra depending on sample type (CM or EVs) and cell of origin (ASCs or DFs). Our data demonstrate that RS can be a valuable tool in the quality and reproducibility assessment of cell-free products to be used in the pipeline of stem cell-derived products for regenerative medicine, as it does not focus on a specific component but rather looks at the true complexity of their composition in which nucleic acids, lipids, carbohydrates, and proteins play specific and key roles.

## Data Availability Statement

The raw data supporting the conclusions of this article will be made available by the authors, without undue reservation.

## Author Contributions

CC, CG, SN, AG, and AB: conceptualization. CC, CG, SN, and AG: methodology. CC, CG, SN, MB, AG, and AB: resources. CC, CG, and AG: data curation and writing and preparation of the original draft. SN, MB, and AB: editing and review of writing. AG and AB: supervision. CC, CG, AG, and AB: project administration. CG, MB, AG, and AB funding acquisition. All authors have read and agreed to the published version of the manuscript.

## Conflict of Interest

The authors declare that the research was conducted in the absence of any commercial or financial relationships that could be construed as a potential conflict of interest.
